# Lactic Acid as a Prophylactic in Gout

**Published:** 1892-10

**Authors:** 


LACTIC ACID AS A PROPHYLACTIC IN GOUT.—Berenger-Feraud (*Journal de Medicine de Paris*, 1892, No 15, p. 181) recommends the employment of lactic acid to prevent the gouty attack. To six hundred grains of the acid is added sufficient water to make a solution of twenty teaspoonfuls, each teaspoonful will thus contain thirty grains. Every morning a teaspoonful of the solution is added to two or three glasses of sweetened water and drank in the course of the day. At the end of twenty days the medication is suspended for ten or twelve days, and then resumed. The treatment should be continued for several years. The remedy is inoffensive, and does not interfere with the digestion or the nutrition.—*Medical Age.*

